# DesMol2, an Effective Tool for the Construction of Molecular Libraries and Its Application to QSAR Using Molecular Topology

**DOI:** 10.3390/molecules24040736

**Published:** 2019-02-18

**Authors:** Inma García-Pereira, Riccardo Zanni, Maria Galvez-Llompart, Jorge Galvez, Ramón García-Domenech

**Affiliations:** 1 Institute of Robotics and Information and Communication Technologies (IRTIC), University of Valencia, 46100 Valencia, Spain; inmaculada.garcia-pereira@uv.es; 2 Molecular Topology and Drug Design Unit, Department of Physical Chemistry, University of Valencia, Avenida V.A. Estelles s/n, Burjassot, 46100 Valencia, Spain; riccardo.zanni@uv.es (R.Z.); maria.galvez@uv.es (M.G.-L.); Jorge.galvez@uv.es (J.G.); 3 Microbiology and Plant Pathology-Unit (CSIC Associated), Department of Microbiology, Faculty of Sciences, University of Malaga, 29071 Malaga, Spain

**Keywords:** DesMol2, topology descriptors, molecular libraries, multilinear regression analysis, linear discriminant analysis, Alzheimer’s disease

## Abstract

A web application, DesMol2, which offers two main functionalities, is presented: the construction of molecular libraries and the calculation of topological indices. These functionalities are explained through a practical example of research of active molecules to the formylpeptide receptor (FPR), a receptor associated with chronic inflammation in systemic amyloidosis and Alzheimer’s disease. Starting from a data(base) of 106 dioxopiperazine pyrrolidin piperazine derivatives and their respective constant values of binding affinity to FPR, multilinear regression and discriminant analyses are performed to calculate several predictive topological-mathematical models. Next, using the DesMol2 application, a molecular library consisting of 6,120 molecules is built and performed for each predictive model. The best potential active candidates are selected and compared with results from other previous works.

## 1. Introduction

Nowadays, one of the biggest challenges in chemistry is to associate a given property (toxicity, biodegradation, pharmacological activity) to a specific compound. For instance, if given a certain chemical structure, being able to determine if it will show a certain experimental property. Researchers, who search for molecules with certain physicochemical, biological or pharmacological properties, have large molecular databases at their disposal. In fact, there are currently more than 144 million chemical compounds cataloged with their CAS registration number [1]. In addition to different commercial molecular databases currently available on the internet (ChemIDplus, DrugBank, Zinc, ChemBank, Specs, Molbase, etc.), along with the information that can be obtained from bibliographic databases (SciFinder, PubMed, Scopus, etc.), researchers can build new molecules thanks to combinatorial chemistry and virtual synthesis. Current software allows the creation of a new molecule in a matter of seconds, without the need of a laboratory to perform the real synthesis. All this requires is the use of quick and effective filtering methodologies and the selection of the most interesting compounds in each field of research and development. QSAR (*Quantitative Structure-Activity Relationship*) modeling techniques represents one of the main computational tools used address this challenge and the process of designing and searching for new compounds. 

The core of any QSAR model lies in molecular descriptors. These descriptors are made up of a series of numerical values that identify the molecule along with a specific physical, chemical or biological property. Depending on the type of information that is parameterized, one can differentiate between constitutional, topological (including topochemical) electrostatic, geometric and quantum indices [2]. 

In particular, the structural characterization of a molecule through its topological indices is an extremely useful tool in QSAR analysis. Molecular topology which converts a molecule into a graph whose vertices are the atoms and whose edges are the bonds, is used to calculate these indices [3]. A proof of molecular topology are the results obtained in the design of new chemical compounds in areas as diverse as cancer [4], malaria [5], Alzheimer [6], mosquito repellents [7], green chemistry [8,9], etc.

Currently, several free access computer tools allow the construction of molecular libraries. The most complete tools are usually desktop applications. Among them are SmiLib v2.0 [10], ChemT [11] and Library Synthesizer [12]. The main weakness when using SmiLib or ChemT is that they do not verify if the SMILES (Simplified Molecular Input Line Entry Specification) codes entered by the user are correct, so that the resulting libraries may contain wrongly identified molecules. On the other hand, Library Synthesizer does not allow the introduction of molecules by means of SMILES code, forcing the user to draw them instead. This can be a very tedious process when the lists of substituents are large. In addition, all three programs have restrictions when introducing the different molecular fragments because they limit the quantity or the combinations that can be made between them. There are also other web applications whose purpose is the construction of molecule libraries. However, their functionalities are limited. For example, cheminfo.org offers a virtual combinatorial library [13], and e-LEA3D has its own combinatorial library design tool [14]. In both cases, the user is forced to draw the different molecular fragments since they do not allow direct entry of SMILES code.

As a result, DesMol2 provides significant advantages over the other leading software. For example, it allows the introduction of more than one base and lists of substituents for each anchor point at a time, and it does not matter where the anchor point of the substituent is placed. It also verifies that the introduced SMILES codes correspond to valid molecular fragments and re-numbers the cycles of the generated molecules to ensure that they are correct. 

In this paper, a web application, DesMol2 [15] is used to combine two functionalities in a single environment. Firstly, it constructs molecular libraries and calculates topological indices. These operations are carried out without the need for any other software, supporting different formats of chemical files, offering the output in compatible formats with classic QSAR analysis programs and automating the creation of new molecules.

One of the biggest advantages of DesMol2 compared to other software with similar functionalities is that it internally checks each SMILES code entered by the user to verify that it is correct. If so, it accepts the fragment to be able to process it later. If it detects an anomaly, it informs the user so any corrections can be made. Similarly, DesMol2 only builds correct molecules, unlike other programs that simply bind SMILES code fragments without verifying whether the result is a well-constructed molecule or not. Our software is using The Chemistry Development Kit (CDK) to carry out this process; this is an open source library with LGPL license.

The steps to follow are:

Using DesMol2, design a complete library of molecules from the SMILES codes of the bases and the substituents defined by Besalú [16].
Calculate with DesMol2 the topological indices of each of the generated molecules.
Build the topological model of activity prediction against the FPR receptors.
Apply the topological model to the library and select the potentially most active molecules.
Compare the results with those reported in other works.

## 2. Results and Discussion

With the help of Statistica software, Lineal Discriminant Analysis (LDA) was performed with the training group, using the grouping variable (see Table S3, Clas(obs)) as the dependent variable and the topological indices calculated by DesMol2 as independent variables.

The discriminant function (DF) selected was:

*DF* = 245.5 – 296.2*C*^1^*χ* + 50.2*D*^4^*χ_pc_* – 0.0034*W* + 4.4*V*4 (1)

N = 85   λ = 0.421   F(4,80) = 27.5   *p* < 0.00001

The DF is statistically significant, both for the equation as a whole, *p* < 0.00001, and for each of the variables that enter the model, *p* < 0.004.

The topological descriptors C^1^χ, D^4^χ_pc_ and V4 provide fundamental topological information related to molecular branching. The model also includes the Wiener index.

When the DF function is applied to each compound, it is classified as active (A) if DF > 0 or inactive (I) if DF < 0. 

Table S3 shows the DF values obtained for each compound together with the activity probability (Prob (A)) and the predicted classification (Clas (pred)) for both the training group and the external validation group. The molecules marked with an asterisk represent classification errors. 

**Figure 1:**
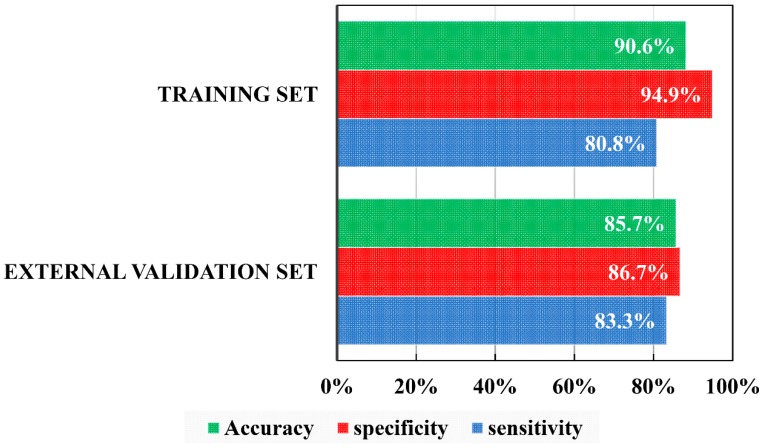
Percentages of correct classification obtained from the LDA (Linear Discriminant Analysis) for each set.

Figure 1 shows the sensitivity (percentage of actives correctly classified), specificity (percentage of inactives correctly classified) and accuracy (percentage global correctly classified) for each data set. In both sets, specificity is higher than 86.5%, which reduces to the maximum the possibility of false assets, while the sensitivity is higher than 80.5%.

**Figure 2:**
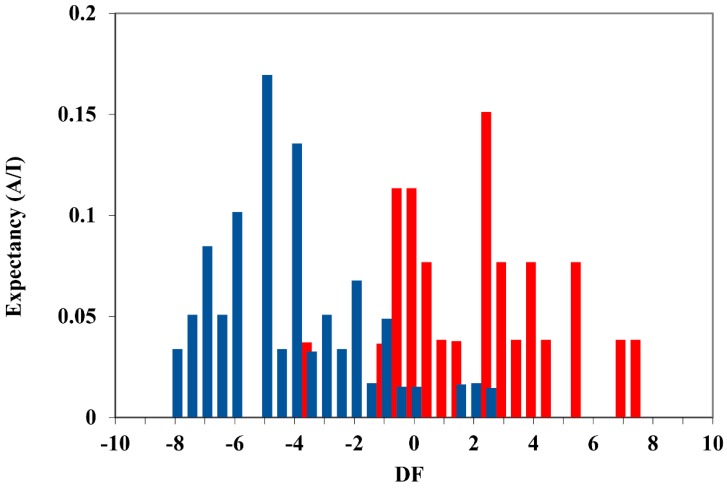
Pharmacological distribution diagram for bonding affinity, k_i_, (red columns represents activity, and blue columns inactivity).

To determine the domain of applicability of the selected discriminant function, DF, the corresponding activity distribution diagram was created, which is shown in Figure 2.

This diagram represents the expectation (probability) of finding active or inactive compounds for different DF values [17]. The red bars represent the active group (Logk_i_ < 2.60) and the blue bars represent the inactive group (Logk_i_ > 2.60). Thus, the useful DF range for classifying the compounds as active is between 0 and 8 such that a compound will be classified as active if DF falls within that range. Values of DF < 0 classify the compound as inactive, while values of DF > 8 or DF < −8 will be outside the domain of applicability of DF and will, therefore, be unclassified.

Once the mathematical model of classification (DF) has been selected, as well as the domain of application, the next step is to apply it to the rest of the previously constructed library. As previously mentioned, the topological indices have been calculated for each molecule with DesMol2. Next, the equation DF is applied to them. All molecules with DF values between 0 and 8 are classified as potentially active. The result was 1403 of a total of 6120, which represents approximately 23% of the bookstore. These 1403 molecules have been classified as active, with a theoretical value of Logk_i_ < 2.60, which represents activity at concentrations lower than 400 nM (see Table S5). To predict the Logk_i_ value of each of these molecules, it will be necessary to perform the corresponding multilinear regression analysis (MLRA). In this case, experimental Logk_i_ values for 48 of the 106 compounds of the working group are available (Logk_i_ values = 4 represent molecules with a concentration higher than 10,000 nM and have been eliminated from the MLRA). Taking the Logk_i_ variable as the dependent and the topological indices as the independent variables, Statistica was used again to perform MLRA analysis. Since the indices of Equation (1) did not correctly predict the LogK_i_ values, which was to be expected as the equation was discriminant in nature and not quantitative, new topological indices were calculated using the Dragon program [18].

The prediction equation selected was:

*Logk_i_* = 76.50 + 0.000033*ww* – 9.50*MAXDN* – 15.68*BELm*5 – 3.18*GGI*10 – 5.12*VEA*1 (2)

**Figure 3:**
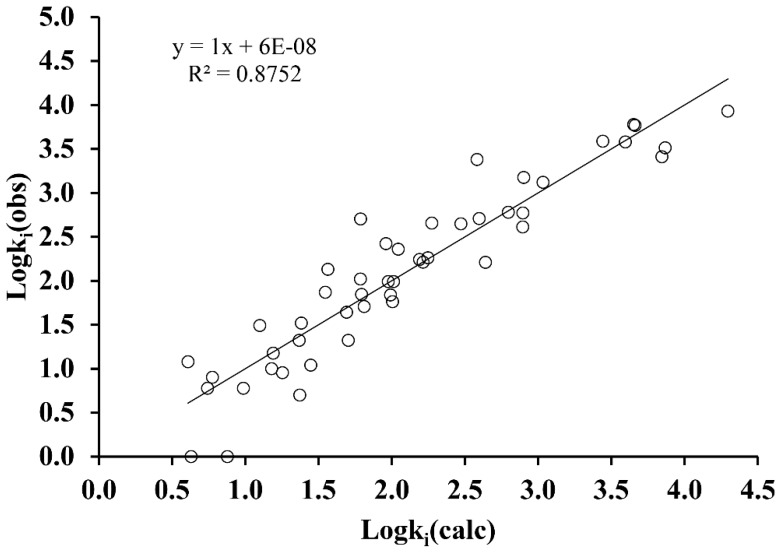
Graphical representation of Logk_i_ (obs) versus Logk_i_ (calc) obtained from Equation (2).

N = 48   R^2^ = 0.875   Q^2^ = 0.841   SEE = 0.376   F = 58.9   *p* < 0.00001

which is a multilinear equation with 5 variables: hyper detour index, ww, [19]; maximal electrotopological negative variation, MAXDN [20] (this descriptor can be related to the nucleophilicity of the molecule [21]); lowest eigenvalue n° 5 of Burden matrix / weighted by atomic masses, BELm5 [22]; topological charge index of order 10, GGI10 [23], and eigenvector coefficient sum from adjacency matrix, VEA1 [22]. All of them were classified as statistically significant (*p* < 0.005) with a high predictive power as indicated by the high value of the correlation coefficient, R^2^ = 0.875, as well as the prediction coefficient obtained in the "leave-one-out" cross-validation, Q^2^ = 0.841. The standard error of estimation, SEE = 0.376, corresponds to a value less than 10% of the interval in which the Logk_i_ property is moving. Table S4 and Figure 3 show the prediction results obtained for each compound and for the set, respectively.

**Figure 4:**
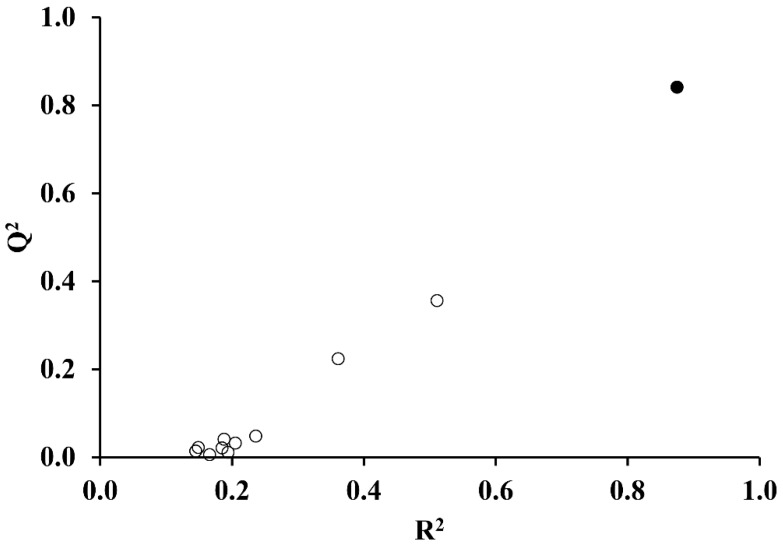
Randomness test performed on the selected regression model, Equation (2).

The randomness test performed at Equation (2) shows excellent stability thereof (see Figure 4).

**Figure 5:**
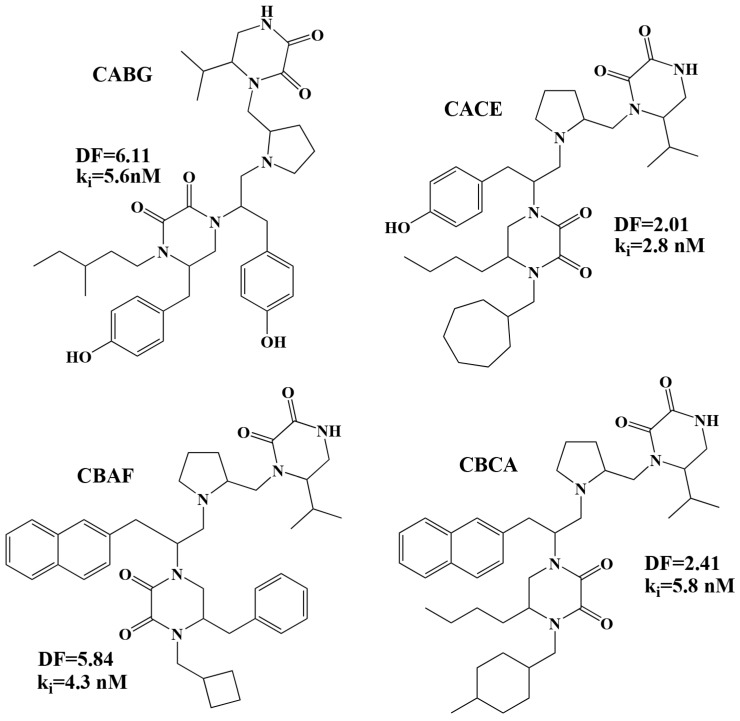
Chemical structure selected by the QSAR model with potential binding activity against the FPR receptor.

In view of the results obtained with the LDA and MLRA, a topological bonding prediction model affinity, k_i_, can be established, consisting of the two functions: Equation (1) and Equation (2). This way, the library generated with DesMol2 (6120 molecules) can be used to select new potential active molecules. The selection criteria were: A DF (Equation (1)) with values between 0 and 8 and Logk_i_ (Equation (2)) with values lower than 2.60, which is equivalent to concentrations lower than 400 nM. Of the 1403 molecules theoretically classified by the DF as active, 785 had a predicted Logk_i_ value < 2.60, which is equivalent to approximately the 13% of the total library molecules (see Table S5, molecules marked in yellow, green and blue). If we limit the activity further and we impose a Logk_i_ < 1.0 (k_i_ < 10 nM), the number of highly active molecules is reduced to 87 (1.4% of the library) (see Table S5, molecules in yellow). Comparing these results with the ones by Besalú [16] using the SSIR (Superposing Significant Interaction Rules) method and the same library, it can be observed that of the 50 molecules selected by the SSIR method as the most active theoretical, 14 of them have been selected with our model with theoretical values of Logk_i_ < 1.0 (CAAA, CABG, CACE, CBAA, CBAF, CBBE, CBBG, CBCA, CBCG, CBCI, CBCL, CBCM, CBCP, CBHC) (see Table S5, molecules in yellow) and 28 with theoretical values of Log k_i_ between 1.0 and 2.0 (CABB, CABD, CACA, CACF, CADC, CADH, CAFC, CAFH, CAHC, CAHH, CAIC, CAIH, CBAD, CBAE, CBBB, CBCD, CBCJ, CBCK, CBCN, CBCO, CBCQ, CBDC, CBDH, CBFC, CBFH, CBHH, CBIC, CBIH) (see Table S5, molecules in green). As can be seen, the most active compounds contain the isopropyl substituent in position R_1_; 4-hydroxybenzyl or 2-naphthylmethyl in R_2_; benzyl, 4-hydroxybenzyl or butyl in R_3_ and different substituents in position R_4_. All this suggests that the substituents indicated above for positions R_1_ and R_2_ are crucial for the design of new active compounds against the FPR receptor. Figure 5 shows the chemical structure of some of the potentially most active molecules.

The next step in the search for new active compounds against RPF would be the synthesis of some of the molecules proposed in Figure 5 and Table S5 (molecules in yellow) and the realization of the corresponding pharmacological tests.

## 3. Materials and Methods

### 3.1. Data Set

The present work consists of a practical exercise using DesMol2 web application for the search of active molecules against the FPR receptors. It starts from the work by Besalú [16] that contains a library of 106 derivatives of dioxopiperazine pyrrolidin piperazine with their respective values of affinity constant of binding for the FPR, k_i_, (see the Table S1 in the Supplementary Material). Besalú presents the SSIR (Superposing Significant Interaction Rules) method as a systematic procedure used to rank series of combinatorial analogues. Table 1 shows the substitution codifications along the four available molecular scaffold sites and Table S1, the codified and activity set of 106 compounds.

### 3.2. Building of the Virtual Molecular Library

The DesMol2 application meets the following functional requirements when building a molecular library:

- The application is capable of generating libraries of molecules by combining different bases, and substituents provided by the user.
- You can enter one or more bases and each of them can have one or more anchor points. For each anchor point, zero or more substituents can be entered. The following syntax indicates the anchor points: [R]. For each substituent, the atom that goes at the anchor point is also indicated.
- All possible combinations are made between bases and substituents.
- The user can graphically visualize each molecular structure, be it a base, a substituent or the result of the combination of both.
- The user is informed when an error occurs when entering the information incorrectly.
- The final result will be a *.smi file, so that each line of it represents a molecule through its code SMILES followed by a unique identifying name. The output file can be used for the calculation of molecular descriptors with other programs such as Dragon [18], PaDel [24], etc.

In the Supplementary Material section, Figure S2 shows a workflow diagram of DesMol2. 

This work starts with a main structure and four anchor points: R_1_, R_2_, R_3_ and R_4_. For each of them, 5, 8, 9 and 17 substituents have been defined, respectively. Therefore, the library generated contains 6120 molecules (see Table S2). To build the library with DesMol2, the SMILES code was obtained from both the base and the substituents (see Table 1), and they were introduced in the DesMol2 application (see Figure S1). Once all the possible combinations between the base and substituents have been made, a *.smi file is obtained that contains the code SMILES of each generated molecule followed by the identification code (see Table S2). The *.smi file generated with the 6120 molecules library will be used to structurally identify each molecule through its corresponding topological indices, also calculated with DesMol2.

In this sense, DesMol2 is a very efficient application, since it is capable of building large libraries of molecules in a few seconds. For the data set chosen in this work, a library of 6120 molecules was built in just 6 s. For its part, the calculation of topological indices has also offered good results. To verify the computational efficiency of our web application, we have calculated the topological indexes of the 6120 molecules with DeMol2, Dragon and PaDel programs. To do this, a group of similar topological indices was selected in the three applications and the average time required for each molecule was obtained: 0.11 s for DesMol2, 0.05 for Dragon and 1.28 for PaDel. Keep in mind that DesMol2 is a web application while Dragon and PaDel are desktop applications. In addition, Dragon is a paid software, while PaDel and DesMol2 are free. Therefore, in view of the results obtained, we can affirm that our software has very competitive response times.

### 3.3. Molecular Descriptors

The DesMol2 application calculates the Kier and Hall topological connectivity indices up the fourth order, ^m^χ_t_ (indexes that evaluate fundamentally the topological assembly of molecules) [25], topological charge indices, Gi, Ji, (which evaluate the intramolecular charge transfer) [23], quotients and differences between the valence and non-valence connectivity indexes (C^m^χ_τ_, D^m^χ_τ_) [26], the Wiener index [27], and a group of constitutional indices (including the number of atoms, degree of branching, etc.) [26]. The recognized formats are: MDL Molfile (*.mol), Smiles (*.smi) and MDL DSfile (*.sdf). The generated topological indexes are formatted as an Excel file that contains two sheets: The first shows the calculated indices for each molecule, while the second contains the file information and unprocessed molecules.

### 3.4. Development of the QSAR Models

In this work, we have used linear discriminant analysis, LDA, and multilinear regression analysis, MLRA. The goal of the linear discriminant analysis (LDA) is to find a linear combination of variables that allow the discrimination between two or more categories or objects. In this case, the data set is formed by 106 molecules distributed in two groups: the active set, A, (Log k_i_ < 2.60, k_i_ < 400 nM) and the inactive set, I, (Log k_i_ > 2.60, k_i_ > 400 nM). LDA has been used to find the grouping descriptors that best discriminate the bonding affinity, k_i_, with the Statistica 9.0 software [28]. The search for the discriminant function is carried out with the training group formed by 80% of the molecules (26 active and 59 inactive). The remaining 20%, selected at random, will serve as an external validation test (6 active and 15 inactive). In addition, as the experimental bonding affinity data of the active and inactive groups are quite unbalanced (26 active and 59 inactive), it is necessary to perform the LDA assigning different weights to each group depending on the existing imbalance. In this study, *p* = 0.306 and *p* = 0.694 were established for the active and inactive groups respectively. Multilinear regression analysis, MLRA, will be used to predict the activity of molecules classified as active by the LDA. Log k_i_ values < 4.0 as the dependent variable and topological indices as independent variables were used respectively. In summary, information is available for 46 molecules. 

The predictability, quality and robustness of the model selected can be verified by means of different types of criteria. In this study two strategies are adopted [29]:

(a) Internal validation or cross-validation with leave-one-out (LOO) analysis. To do this, one compound of the set is extracted, and the model is recalculated using as a training set the remaining N-1 compounds. The property is then predicted for the removed element. This process is repeated for all the compounds of the set, obtaining a prediction for each of them. From the residual values obtained, the prediction coefficient, R^2^(cv) or Q^2^ is determined.
(b) Data randomization or Y-scrambling. A randomization test can be analyzed to identify the possible existence of fortuitous correlations [30]. To do this, the values of the property of each compound are randomly permuted and linearly correlated with the topological descriptors.

## 4. Conclusions

DesMol2 software is presented as a tool available to researchers for the construction of molecular libraries and their subsequent implementation in QSAR analysis. Using SMILES code, large libraries can be designed consisting of one or more structural starting bases and different substituents or anchor points. The output format of the generated library is prepared for the calculation of topological descriptors, either with the DesMol2 itself or other existing software in the market and applied in the QSAR model search. DesMol2 is an open-access web application that, unlike other software with similar functionalities, provides great flexibility when introducing molecular fragments and always verifies the validity of both the introduced SMILES codes and the resulting molecules after making all possible combinations.

## Figures and Tables

**Table 1:**
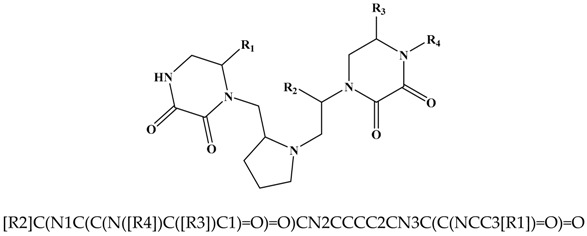
Base structure and substituents used in the construction of the molecular library.

Code	R1	R2	R3	R4
**A**	2-naphthylmethyl	4-hydroxybenzyl	benzyl	4-Methyl-1-cyclohexyl-methyl
	[R]C1=CC2=CC=CC=C2C=C1	OC1=CC=C(C=C1)[R]	[R]C1=CC=CC=C1	[R]C1CCC(C)CC1
**B**	propyl	2-naphthylmethyl	4-hydroxybenzyl	Cyclohexylpropyl
	CC[R]	[R]C1=CC2=CC=CC=C2C=C1	OC1=CC=C(C=C1)[R]	[R]CCC1CCCCC1
**C**	isopropyl	cyclohexyl	butyl	Cyclohexylmethyl
	[R](C)(C)	C1CCCCC1[R]	[R]CCC	[R]C1CCCCC1
**D**	butyl	propyl	propyl	Cyclopentylmethyl
	[R]CCC	CC[R]	CC[R]	[R]C1CCCC1
**E**	benzyl	hydroxymethyl	S-phenyl	Cycloheptylmethyl
	[R]C1=CC=CC=C1	O[R]	[R]1=CC=CC=C1	[R]C1CCCCCC1
**F**		butyl	butyl	Cyclobutylmethyl
		[R]CCC	[R]CCC	[R]C1CCC1
**G**		benzyl	cyclohexyl	3-Methylpentyl
		[R]C1=CC=CC=C1	C1CCCCC1[R]	[R]CC(C)CC
**H**		isobutyl	benzyl	2-Biphenyl-4-yl-ethyl
		CC(C)[R]	[R]C1=CC=CC=C1	[R]CC(C=C1)=CC=C1C2=CC=CC=C2
**I**			propyl	4-Tert-butyl-cyclohexylmethyl
			CC[R]	[R]C1CCC(C(C)(C)C)CC1
**J**				2-(3-Methoxyphenyl)-ethyl
				[R]CC1=CC(OC)=CC=C1
**K**				2-(4-Isobutylphenyl)-propyl
				[R]C(C1=CC=C(CC(C)C)C=C1)C
**L**				m-Tolylethyl
				[R]CC1=CC(C)=CC=C1
**M**				p-Tolylethyl
				[R]CC1=CC=C(C)C=C1
**N**				2-(4-Methoxyphenyl)-ethyl
				[R]CC1=CC=C(OC)C=C1
**O**				2-(4-Ethoxyphenyl)-ethyl
				[R]CC1=CC=C(OCC)C=C1
**P**				Phenethyl
				[R]CC1=CC=CC=C1
**Q**				3-(3,4-Dimethoxyphenyl)-propyl
				[R]CCC1=CC=C(OC)C(OC)=C1

## Supplementary Materials

All TIs used in the present study along with their values for every compound (training, test, and molecular library) are shown in the supporting information section.
